# Practice of Dentistry in the State of South Carolina

**Published:** 1875-04

**Authors:** 


### EDITORIAL, ETC.

---

*Practice of Dentistry in the State of South Carolina.*—An Act to Regulate the Practice of Dentistry and protect the people against Empiricism, in relation thereto, in the State of South Carolina.

*Be it enacted by the Senate and House of Representatives of the State of South Carolina, now met and sitting in General Assembly, and by the authority of the same:* Section 1. That from and after the passage of this Act, it shall be unlawful for any person or persons to engage in the practice of dentistry in the State of South Carolina, unless said person or persons shall receive a diploma from the faculty of some dental college, duly incorporated under the laws of this or some other State of the United States, or foreign Government, in which is annually delivered in good faith, a full course of lectures and instructions in dentistry, or shall have obtained a license from a Board of Dentists, duly authorized and appointed by this Act to issue such license.

SEC. 2. It shall be the duty of the South Carolina State Dental Association, at the next annual meeting thereof after the passage of this Act, to elect a Board of Examiners, to consist of five members, to be known by the title of the Board of Dental Examiners in the State of South Carolina. The members of this Board shall, at the first election, be elected for terms of one, two, three, four and five years, respectively, or until their successors shall have been elected. And it shall be the duty of the South Carolina State Dental Association, at each subsequent annual meeting thereof, to elect a person for the term of five years to fill the place of the member of the Board whose term of office shall at that time expire, and also to fill such vacancies in the Board as may have occurred during the year. And if at any regular meeting of the Board, any member or members shall fail to be present, the South Carolina State Dental Association may, at its discretion, declare the office of such absentees to be vacated, and may proceed to elect a new member or members for the unexpired term of such person or persons, or it may elect a member or members to fill temporarily the place or places of such absentees. This Board shall be organized by the election of a President and Secretary.

SEC. 3. It shall be the duty of the Board of Examiners to meet annually at the time and place of meeting of the South Carolina State Dental Association, giving thirty days' notice in the public newspapers published in not less than three different places in the State, viz: One in Charleston, one in Columbia and one in Greenville, of such annual meeting. Secondly, to prescribe a course of reading for those who study dentistry under private instructions. Thirdly, to grant a license to any applicant who shall furnish satisfactory evidence of having graduated, and received a diploma from any incorporated dental college in good standing with the profession, without fee, charge or examination. Fourthly, to grant license to all applicants who undergo a satisfactory examination. Fifthly, to keep a book in which shall be registered all persons licensed to practice dentistry in the State of South Carolina. The expenses of said license shall be fifteen dollars, to be paid by the licensee. And that all persons who do now hold, or may hereafter hold, a license to practice dentistry in this State, shall become a member of the South Carolina State Dental Association immediately upon obtaining said license; *Provided,* He shall be allowed to waive his right of membership.

SEC. 4. That the books so kept shall be a book of record, and a transcript from it, certified by the officer who has it in keeping with the common seal, shall be evidence in any Court of the State.

SEC. 5. That three members of said Board shall constitute a quorum for the transaction of business, and should a quorum not be present on the day appointed for their meeting, those present may adjourn from day to day until a quorum is present.

SEC. 6. That one member of said Board may grant a license to an applicant to practice until the next regular meeting of the Board, when he shall report the fact, at which time the temporary license shall not be granted by a member of the Board after the Board has rejected the applicant.

SEC. 7. That every dentist in this State be required to keep a record of all cases treated in his practice, in accordance with a form to be designated by the South Carolina State Dental Association, and furnish his patient with a copy of the same, if so desired, by the patient.

SEC. 8. That any person who shall, in violation of this Act, practice dentistry in the State of South Carolina for fee or reward, shall be liable to indictment, and on conviction shall be fined not less than fifty or more than three hundred dollars: *Provided,* That nothing in this Act shall be so construed as to prevent any person from extracting teeth.

SEC. 9. That on trial of such indictment, it shall be incumbent on the defendant to show that he has authority under the law to practice dentistry, to exempt himself from such penalty.

SEC. 10. That all fines collected shall inure to the educational fund of the County where the offender resides.

SEC. 11. That those who have been in the regular practice of dentistry in the State prior to the passage of this Act are exempt from the provisions of the same, except Section 7 of this Act.

SEC. 12. That the South Carolina State Dental Association is hereby made a body politic, and corporate, shall have and use a common seal, sue and be sued, plead and be impleaded, and be empowered to make all necessary by-laws not inconsistent with the State laws and Constitution.

SEC. 13. That all Acts or parts of Acts inconsistent with this Act be, and the same are hereby, repealed.

The above Act has received the Signature of the Governor, and is now a law of the State.

---

